# Using tissue clearing and light sheet fluorescence microscopy for the three‐dimensional analysis of sensory and sympathetic nerve endings that innervate bone and dental tissue of mice

**DOI:** 10.1002/cne.25582

**Published:** 2024-01-27

**Authors:** Jenny Thai, John‐Paul Fuller‐Jackson, Jason J. Ivanusic

**Affiliations:** ^1^ Department of Anatomy and Physiology University of Melbourne Parkville Victoria Australia

**Keywords:** bone, femur, iDISCO, mandible, nerves, sensory, sympathetic, tissue clearing

## Abstract

Bone and dental tissues are richly innervated by sensory and sympathetic neurons. However, the characterization of the morphology, molecular phenotype, and distribution of nerves that innervate hard tissue has so far mostly been limited to thin histological sections. This approach does not adequately capture dispersed neuronal projections due to the loss of important structural information during three‐dimensional (3D) reconstruction. In this study, we modified the immunolabeling‐enabled imaging of solvent‐cleared organs (iDISCO/iDISCO+) clearing protocol to image high‐resolution neuronal structures in whole femurs and mandibles collected from perfused C57Bl/6 mice. Axons and their nerve terminal endings were immunolabeled with antibodies directed against protein gene product 9.5 (pan‐neuronal marker), calcitonin gene–related peptide (peptidergic nociceptor marker), or tyrosine hydroxylase (sympathetic neuron marker). Volume imaging was performed using light sheet fluorescence microscopy. We report high‐quality immunolabeling of the axons and nerve terminal endings for both sensory and sympathetic neurons that innervate the mouse femur and mandible. Importantly, we are able to follow their projections through full 3D volumes, highlight how extensive their distribution is, and show regional differences in innervation patterns for different parts of each bone (and surrounding tissues). Mapping the distribution of sensory and sympathetic axons, and their nerve terminal endings, in different bony compartments may be important in further elucidating their roles in health and disease.

## Graphical abstract

We modified the iDISCO/iDISCO+ tissue clearing protocol and used light sheet microscopy to image high‐resolution neuronal structures in whole femurs and mandibles collected from C57Bl/6 mice. We report high‐quality immunolabeling of the axons and nerve terminal endings for both sensory and sympathetic neurons, describe their projections through full 3D volumes of bone, highlight how extensive their distribution is, and show regional differences in innervation patterns for each.

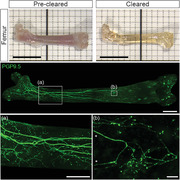

## 1 INTRODUCTION

Bone and dental tissues are richly innervated by sensory and sympathetic nerve fibers (Byers, 1984; Castaneda‐Corral et al., 2011; Chartier et al., 2018; Hildebrand et al., 1995; Mach et al., 2002; Nencini et al., 2017; Paik et al., 2009; Ramieri et al., 1990; Thai et al., 2020). Together, they have important roles in pain transmission (Furusawa, 1970; Nencini & Ivanusic, 2017), regulation of bone homeostasis (Ding et al., 2010; Offley et al., 2005), bone remodeling and healing (Corr et al., 2017; Elefteriou et al., 2005; Ma et al., 2013; Wang et al., 2010), regulation of the vasculature (Bjurholm, Kreicbergs, Terenius, et al., 1988), hematopoiesis (Katayama et al., 2006; Yamazaki et al., 2011), osteogenesis/odontogenesis (Chartier et al., 2018; Sisask et al., 2013; Zhao et al., 2014), and/or innocuous dental mechanosensitivity (Byers, 1985; Kannari, 1990; Kannari et al., 1991; Trulsson, 2006; Trulsson et al., 1992). Furthermore, various resident cell types in hard tissue (e.g., osteoblasts/odontoblast, osteoclasts, and chondrocytes) are in close proximity to the nerve endings and express the receptors for signaling molecules secreted by nerves, including calcitonin gene–related peptide (CGRP) and substance P (Cornish et al., 2001; Ishizuka et al., 2005; Jones et al., 2004; Togari et al., 1997; Utagawa et al., 2023), or noradrenaline (Elefteriou et al., 2005, 2014). Characterization of the morphology, molecular phenotype, and distribution of nerves that innervate bone and dental tissues is important to inform their function in both health and disease.

Our current understanding of the distribution of nerves that innervate hard tissue has relied heavily on conventional histological analysis of thin sections (Chartier et al., 2018; Widbiller et al., 2021). Although histological studies provide valuable and robust data, it is difficult to appreciate and comprehensively analyze dispersed neuronal projections due to the loss or distortion of important structural information when reconstructing three‐dimensional (3D) images from multiple sections (Lorenz et al., 2021; Lyroudia et al., 1993). Furthermore, 3D reconstruction is often difficult, time‐consuming, and can cause inaccuracies in the images produced due to the variability of staining between sections and the introduction of artifacts. Recent advances in optical clearing techniques and light sheet fluorescence microscopy have allowed for the 3D visualization of neuronal structures in intact, complete tissue at cellular resolution (Richardson et al., 2021; Tian et al., 2021; Ueda et al., 2020), and there has been significant recent interest in applying these approaches to hard tissues like bone (Jing et al., 2019; Lorenz et al., 2021; Utagawa et al., 2023).

Optical clearing enables deep‐tissue imaging by reducing light scattering and absorption and by matching the refractive index of the tissue to that of the background medium (Ueda et al., 2020). A major obstacle in imaging optically cleared calcified tissue, such as bone and dental tissues, is the presence of calcium phosphate minerals that greatly obscure and scatter light (Jing et al., 2019). Therefore, decalcification is crucial for the complete transparency of hard tissue. Furthermore, bone and bone marrow are rich in collagen and adipose tissue, and the bone marrow houses a plethora of pigmented cells that absorb light such as heme‐containing red blood cells (Jing et al., 2019). The dental pulp, which contains blood vessels and nerves, is also protected by the enamel, one of the hardest and most mineralized tissues in the body (Cho et al., 2010). Therefore, optical clearing of hard tissue requires selecting appropriate clearing agents for sample transparency to allow for enhanced imaging depth.

One optical clearing method that has been developed and adapted over the last decade is immunolabeling‐enabled imaging of solvent‐cleared organs (iDISCO/iDISCO+) (Renier et al., 2016, 2014). iDISCO/iDISCO+ is a simple and inexpensive technique that enables deep penetration of antibodies and provides a stable fluorescence signal over time. In the present study, we used a modified version of the iDISCO/iDISCO+ clearing protocol (Fuller‐Jackson et al., 2021), optimized here for hard tissue in mice, to examine the innervation of bone and dental tissues in 3D.

## 2 MATERIALS AND METHODS

### 2.1 Animals

**FIGURE 1:**
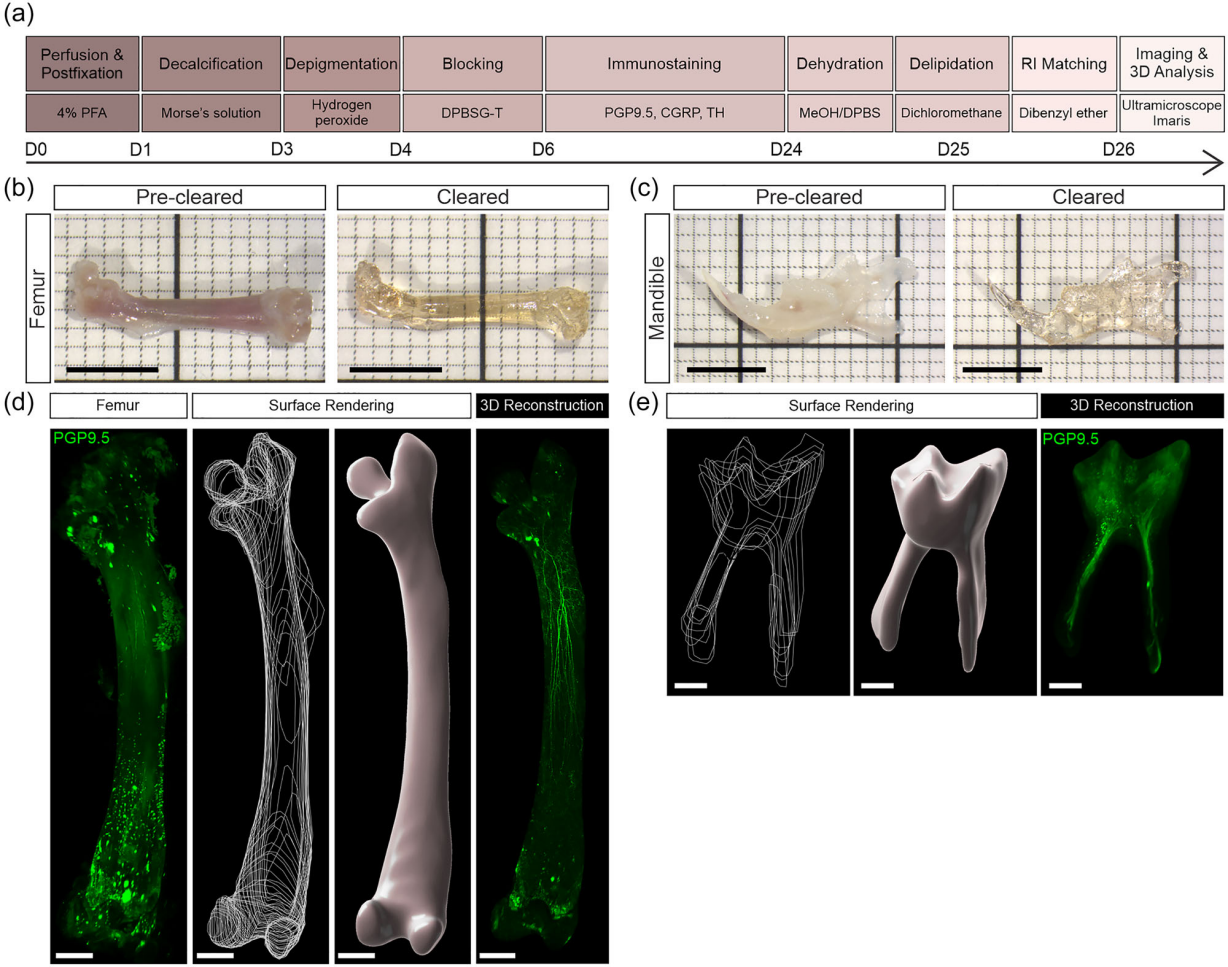
Modified immunolabeling‐enabled imaging of solvent‐cleared organs (iDISCO/iDISCO+) tissue clearing protocol achieves transparency of mouse femur and dental tissue. (a) Schematic diagram of the tissue clearing procedure for the three‐dimensional (3D) visualization of nerve fibers. (b and c) Whole mouse femur (b) and mandible (c) imaged before and after clearing (scale bars, 5 mm). (d and e) A 3D rendered surface of the bone marrow (d, scale bars, 1000 μm) and dental pulp (e, scale bars, 200 μm) tissue using manually drawn boundaries (20–30 μm apart) to view nerve fibers immunolabeled for protein gene product 9.5 (PGP9.5).

Decalcification, sample preparation, immunostaining, and tissue clearing were performed on femurs and mandibles taken from naïve, male and female, 7‐ to 17‐week‐old C57BL/6 mice (*n* = 11) (Figure 1). To minimize the total number of animals we used, we often collected and processed two femurs, and/or two halves of the mandible, from each animal. All experiments conformed to the Australian National Health and Medical Research Council code of practice for the use of animals in research and were approved by the University of Melbourne Animal Experimentation Ethics Committee.

### 2.2 Tissue collection and decalcification

Mice were anesthetized with ketamine/xylazine (ketamine 100 mg/kg, xylazine 15 mg/kg; i.p.) and perfused via the left ventricle with 100 mL heparinized saline containing 1% sodium nitrate followed by 100 mL of 4% paraformaldehyde (PFA) in 0.1 M phosphate buffer (pH 7.4). Femurs and mandibles were removed, placed onto a Petri dish containing chilled 0.1 M phosphate‐buffered saline (PBS) solution, and carefully cleaned of surrounding soft tissue. Bones were postfixed in 4% PFA with rotation at 4°C for 24 h. Samples were then washed three times in PBS and decalcified in Morse's solution (22.5% formic acid, 10% tri‐sodium citrate in Milli‐Q H_2_O) for 30–40 h, with rotation at room temperature. After decalcification, samples were washed six times (15 min each wash) in Dulbecco's PBS (DPBS, Gibco) and stored for up to 2 days at 4°C before proceeding with depigmentation.

### 2.3 Depigmentation

All steps from this point were performed on a rotator and carried out at room temperature unless specified otherwise. Samples were gradually dehydrated by successive immersion in 50%, 80%, and 100% methanol in DPBS for 1.5 h each. Samples were then bleached in 6% hydrogen peroxide in methanol at 4°C overnight (in the dark without agitation). The next day, tissues were gradually rehydrated by successive immersion in 100%, 100%, 80%, and 50% methanol in DPBS for 1.5 h each, and then in DPBS for 1.5 h. Samples were then processed for immunostaining.

### 2.4 Immunostaining

Nerves in samples were immunolabeled using antibodies raised against protein gene product 9.5 (PGP9.5; pan‐neuronal marker) (Christensen et al., 1993; Imai et al., 1994), tyrosine hydroxylase (TH; sympathetic neuronal marker) (Bjurholm, Kreicbergs, Terenius, et al., 1988; Imai et al., 1997), and CGRP (peptidergic nociceptive marker) (Bjurholm, Kreicbergs, Brodin, et al., 1988; Gibbins et al., 1985; Hill & Elde, 1988; Imai et al., 1997). Details of the primary and secondary antibodies are provided in Table 1. All antibodies were diluted in DPBS containing 0.2% gelatine, 0.5% Triton X‐100, 0.01% thimerosal (DPBSG‐T), and 0.1% saponin. Following depigmentation, samples were blocked in DPBSG‐T for 36 h. They were then incubated in primary antibody (Table 1) solution for 11 days at 37°C with gentle orbital shaking. Samples were washed six times in DPBS containing 0.5% Triton X‐100 (DPBS‐T) and then incubated in secondary antibody (Table 1) solution for 7 days at 37°C with gentle orbital shaking. Following another six washes with DPBS‐T, samples were transferred to 4 mL glass vials with a silicon‐coated cap (Sigma‐Aldrich).

**TABLE 1:** Details of the primary and secondary antibodies used in this study.

Primary antibody	Immunogen	Manufacturing details	Specificity/Characterization	Dilution
Calcitonin gene–related peptide (CGRP)	Synthetic CGRP (rat) conjugated to KLH	Sigma‐Aldrich; rabbit polyclonal; Cat C8198, RRID: AB_259091	Lorenzo et al. (2008), Yen et al. (2006)	1:1000
Protein gene product 9.5 (PGP9.5)	15 amino synthetic peptide located near the C‐terminus of human PGP9.5 (UCHL1)	Cedarlane Laboratories; rabbit polyclonal; Cat CL7756AP, RRID: AB_2792979	Doran et al. (1983)	1:500
Tyrosine hydroxylase (TH)	Denatured TH from rat pheochromocytoma	Millipore; rabbit polyclonal; Cat AB152, RRID: AB_390204	Brown et al. (2011)	1:1000
**Secondary antibody**		**Manufacturing details**		**Dilution**
Donkey anti‐Rabbit Alexa Flour 647		Invitrogen; Cat A31573		1:1000

Abbreviation: KLH, keyhole limpet hemocyanin.

### 2.5 Tissue clearing

After immunolabeling, tissues were gradually dehydrated by successive immersion in 20%, 40%, 60%, 80%, 100%, and 100% methanol in DPBS for 1 h each. They were then incubated overnight in 66% dichloromethane (DCM) and 33% methanol. The following day, samples were incubated twice in 100% DCM for 30 min each, and then in dibenzyl ether (DBE) until cleared (2 h). They were stored in fresh DBE at room temperature in the dark. Samples were transferred to ethyl cinnamate (ECi) prior to imaging and returned to DBE for long‐term storage.

### 2.6 Antibody specificity and characterization

The anti‐CGRP antibody (Sigma‐Aldrich, product code C8198, RRID: AB_259091) was raised in rabbit against synthetic rat CGRP conjugated to keyhole limpet hemocyanin (manufacturer's information). This antibody shows no cross‐reactivity with other peptides, except with rat and human CGRP, and human β‐CGRP. Staining of sensory nerve terminals in rat glabrous skin was abolished when the antibody was preadsorbed with rat CGRP (Yen et al., 2006). When the primary antibodies were replaced with preimmune serum from the same source species, no staining was observed (Lorenzo et al., 2008; Yen et al., 2006).

The anti‐PGP9.5 antibody (Cedarlane Laboratories, product code CL7756AP, RRID: AB_2792979) was raised in rabbit against synthetic peptide located near the C‐terminus of human PGP9.5 and labels a single band at 27 kDa in Western blots of human cerebellum, and mouse and rat brain (manufacturer's information). Immunostaining in rat brain was completely abolished following preadsorption with purified PGP9.5 (Doran et al., 1983).

The anti‐TH antibody (Millipore, product code AB152, RRID: AB_390204) was raised in rabbit against denatured TH from rat pheochromocytoma and labels a single band at 62 kDa in Western blots of PC12 lysates (manufacturer's information). No staining was observed in mutant mice that are deficient in subsets of midbrain dopamine neurons (Brown et al., 2011).

### 2.7 Stereomicroscope and light sheet fluorescence microscope imaging

Macroscopic samples were imaged with a stereomicroscope (SZX7 Zoom Stereo Microscope, Olympus) equipped with an Axiocam 105 color camera (Zeiss) and a 0.5×/0.05 objective lens (DFPL, Olympus) (Figure 1). Images were captured using Zen Blue software (Version 3.1, Zeiss).

Cleared samples were imaged with a light sheet fluorescence microscope (UltraMicroscope II, Miltenyi Biotec) equipped with a Neo sCMOS camera (Andor, 2560 × 2160 pixel size) and a 2×/0.5 (MVPLAPO, Olympus) objective lens attached to a zoom body with a magnification range of 0.63×–6.3× (MVX‐10, Olympus). For high‐resolution imaging of the dental pulp, some samples were imaged with a 12×/0.53 (LVMI PLAN, LaVision) fixed objective lens. For image acquisition, cleared samples were mounted onto the correctly sized sample holder with super glue and immersed in ECi in a quartz cuvette. To visualize labeling with the Alexa Fluor 647 fluorophore, a 639 nm laser diode (LASOS) was used, and the emitted wavelength was detected with the Cy5 emission filter (680/30 nm). Samples were illuminated from a single‐side using three combined light sheets with a thickness of 5 μm and no dynamic focusing. Optical *z*‐stacks were generated using a step size of 2 μm, with 100–200 ms exposure per step, to examine nerve endings through the full thickness of cleared samples. Mosaic acquisitions were performed with 10% overlap. Images were captured using Imspector Pro Software (Version 5.1.328, Abberior Instruments).

### 2.8 Image processing

Individual tagged image file (.tif) images were converted into Imaris file format (.ims) using the Imaris File Converter (Version 9.9.1, Bitplane). Mosaic images were fused using the Imaris Stitcher x64 (Version 9.3.1, Bitplane), and 3D visualization and segmentation were performed using Imaris x64 software (Version 9.9.1, Bitplane). Segmentation of the bone marrow and dental pulp from the decalcified bone was performed using the Imaris “Surface” module. This was an important step because the autofluorescence of mineralized bone and surrounding muscle/ligaments often obscured labeling deep in bone. The anatomical boundary was manually defined using multiple cross‐sectional drawings, 20–30 μm apart, to create a 3D surface render of the bone marrow or dental pulp tissue (Figure 1). A mask of the rendered surface was then performed to produce a new artificial channel showing nerve fibers only within the selected region of interest. Tracing of some neuronal axons and nerve terminal endings was performed using the Imaris “Filament Tracer” module. Projections were automatically traced using the “Autopath (no loops)” algorithm. The final filament tracing was checked and corrected semi‐automatically. The total length of filament, number of branching points, and terminal endings are reported to provide an example of what can be achieved using this approach, but we did not quantify all labeling of axons and their terminal endings throughout each tissue in this study. Adjustments to the brightness and contrast were made to individual images for better viewing. All figures were prepared on CorelDRAW software (CorelDRAW Graphics Suite, Corel Corporation) and are presented as 3D images or maximum intensity projections on Imaris. Maximum intensity projections were pseudo‐colored green. Movies were generated on Imaris with a frame rate of 24 frames per second.

## 3 RESULTS

The iDISCO/iDISCO+ tissue clearing protocol reported here enabled visualization of nerve fibers in mouse femur and mandible immunolabeled with antibodies directed against PGP9.5 (*n* = 5 femur, *n* = 4 mandibles), CGRP (*n* = 5 femurs, *n* = 4 mandibles), and TH (*n* = 5 femurs, *n* = 5 mandibles). In this study, we have focused on the innervation of the marrow cavity of the femur, and the tooth pulp and mandible, rather than their periostea and/or surrounding tissues, which were mostly removed during tissue processing. We report some findings for the periosteum and surrounding tissues when present in our preparation. This was particularly relevant for the mandibular samples.

### 3.1 PGP9.5‐immunolabeled nerve fibers in the marrow cavity of the femur

**FIGURE 2:**
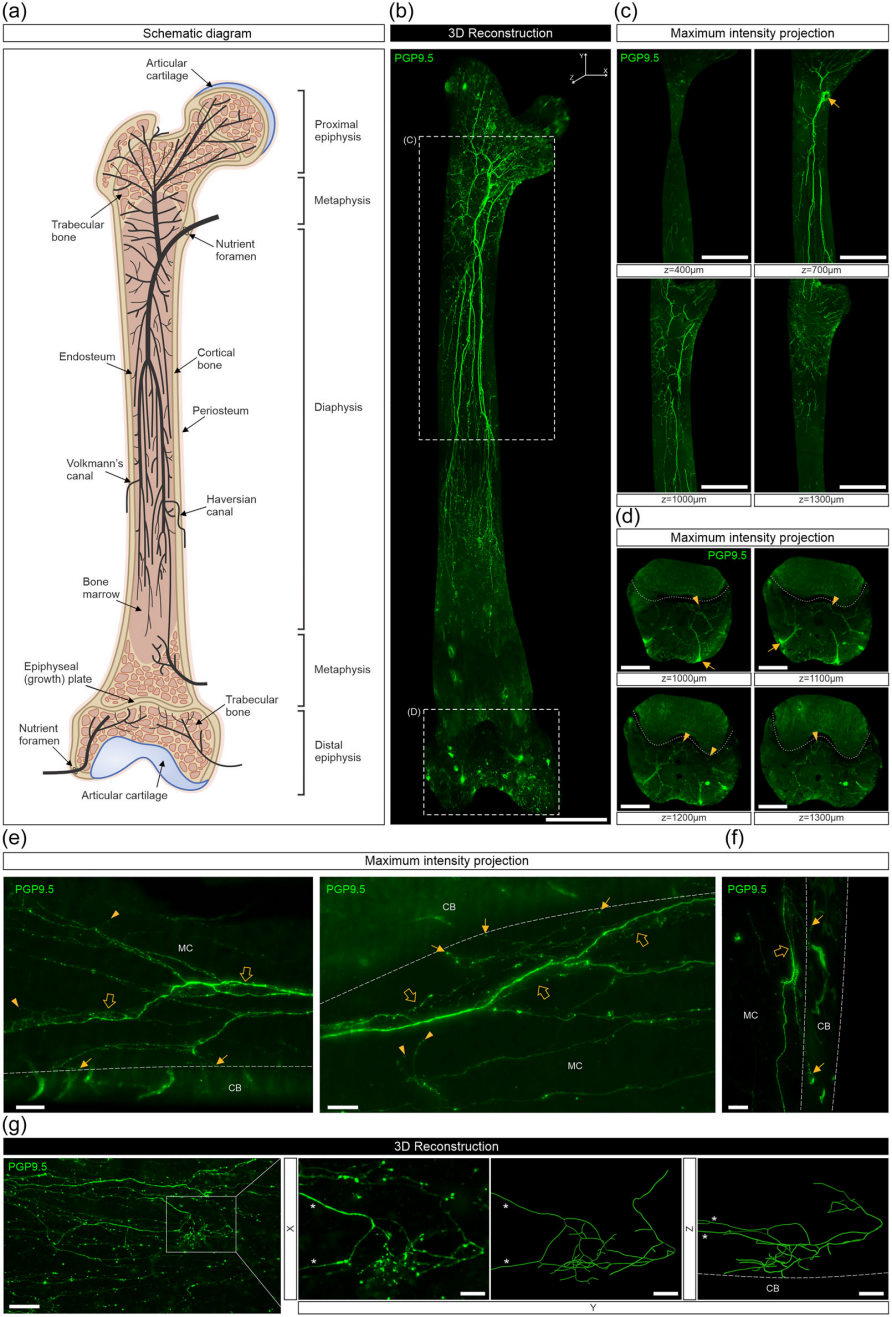
Whole‐tissue immunolabeling and three‐dimensional (3D) fluorescence imaging to visualize protein gene product 9.5 (PGP9.5)‐labeled nerve fibers and their endings in the mouse femur. (a) A simplified schematic representation of the neuronal distribution in the mouse femur. (b) A mosaic stitched, 3D reconstructed image of the bone marrow after surface rendering (scale bars, 1000 μm). (c) Insets show 300 μm maximum intensity *z*‐projections of PGP9.5‐labeled nerve fibers indicated in (b). Arrow indicates the entry site of a nerve bundle into the marrow cavity (scale bars, 1000 μm). (d) Insets show 200 μm *z*‐projections of nerve fibers in the trabecular bone indicated in (b). Nerve bundles enter through the distal epiphysis (arrows), where they branch out and terminate in the marrow cavity (arrowheads). Dotted lines indicate the growth plate (scale bars, 500 μm). (e) 300 (left) and 200 μm (right) *z*‐projections of nerve fibers imaged at higher power, showing PGP9.5‐labeled axons wrapping around or running parallel to blood vessels (open arrows) and branching out and terminating near the endosteum (arrows) or in the marrow cavity (arrowheads) (scale bars, 100 μm). (f) A 100 μm *z*‐projection showing PGP9.5‐labeled axons following vessels in the marrow cavity (open arrows) and running through canals in the cortical bone (arrows) (scale bar, 100 μm). (g, left) A 3D reconstructed image of PGP9.5‐labeled nerve fibers taken in the lower diaphysis (scale bar, 200 μm). (Right) Insets show a 3D reconstruction and tracing of a cluster of complex nerve endings indicated in the white box viewed from the front (*XY*‐plane; left) and side (*YZ*‐plane; right). Asterisks indicate parent axons (scale bars, 50 μm). Dotted lines indicate cortical bone. CB, cortical bone; MC, marrow cavity.

PGP9.5 is a pan‐neuronal marker that indiscriminately labels axons and nerve terminal endings of any type. In the femur, PGP9.5‐labeled nerve bundles entered the marrow cavity via nutrient foramina located at the proximal metaphysis (Figure 2a–c) and the distal epiphysis of the femur (Figure 2a,b,d) (Video S1). The nerves that entered the marrow cavity at the proximal end of the femur branched extensively into the trochanter and through the neck of the femur above (Figure 2c), and into the marrow cavity of the diaphysis below (Figure 2b,c) (Video S1). The branches that ran down through the diaphysis often ran in parallel and terminated over long distances, some extending all the way down to the distal metaphysis above, but not crossing the growth plate (Figure 2a,b, Video S1). The nerves that entered through the distal epiphysis terminated in the marrow cavity of the subchondral bone below the growth plate (Figure 2d). Nerve fibers often followed blood vessels through the marrow cavity, where they could be seen terminating with a classic corkscrew morphology around blood vessels (Figure 2e, open arrows) or as free endings away from blood vessels (Figure 2e, arrowheads). Some of the free endings had *en passant* varicosities along their length and some did not, and they often ended with single bulbar endings (Figure 2e). The innervation density decreased lower in the femur and was least in the lower third of the diaphysis and the distal metaphysis. As the nerve bundles ran down the femur, they often gave branches that projected laterally toward the endosteum and terminated near cortical bone (Figure 2e, arrows). Some axons communicated with Volkmann's and Haversian canals in the cortical bone, *en route* to or from the periosteum (Figure 2f, arrows). On occasions, we observed clusters of very complex endings associated with the termination of a single branched axon (Figure 2g, Video S2). These were mostly located in the mid‐to‐lower diaphysis region. An example filament trace of a PGP9.5‐labeled complex ending had a total length of ∼3729 μm, with 43 branching points and 25 terminal endings (Figure 2g).

### 3.2 CGRP‐immunolabeled nerve fibers in the marrow cavity of the femur

**FIGURE 3:**
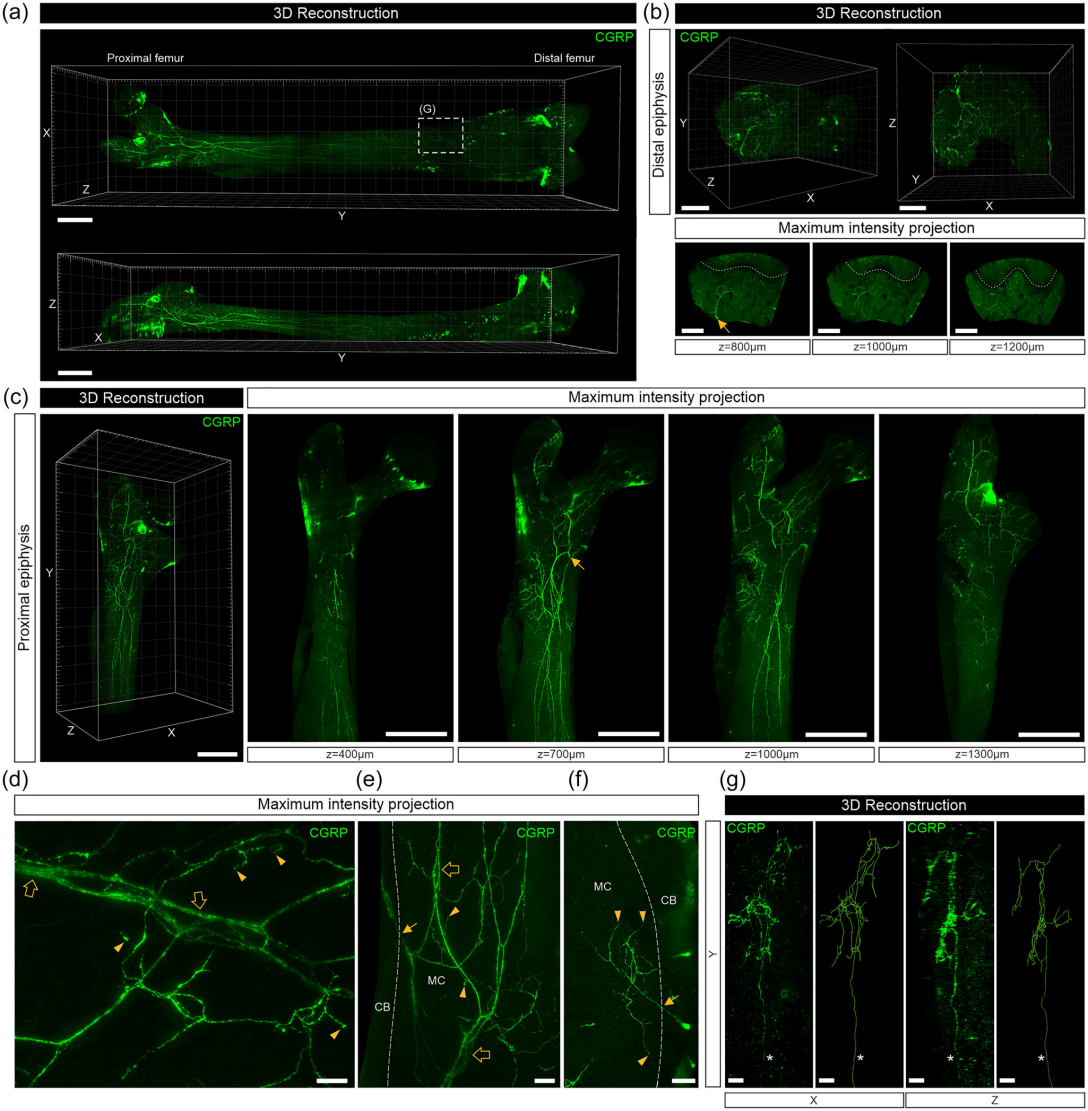
Whole‐tissue immunolabeling and three‐dimensional (3D) fluorescence imaging to visualize calcitonin gene–related peptide (CGRP)‐labeled nerve fibers and their endings in the mouse femur. (a) Mosaic stitched, 3D reconstructed images of the bone marrow after surface rendering (scale bars, 1000 μm). (b, above) 3D‐reconstructed images of the distal femoral epiphysis. (b, below) Insets show 500 μm maximum intensity *z*‐projections of a nerve bundle (arrow) entering and branching out in the epiphysis. Dotted lines indicate the growth plate (scale bars, 500 μm). (c, left) A 3D‐reconstructed image of the proximal femoral epiphysis and diaphysis. (c, right) Insets show 500 μm *z*‐projections of a CGRP‐labeled nerve bundle (arrow) entering the marrow cavity and branching out into the femoral neck and down the diaphysis (scale bars, 1000 μm). (d) A 400 μm *z*‐projection of nerve fibers imaged at higher power, showing CGRP‐labeled nerve axons running parallel to blood vessels (open arrows) and branching out into the marrow cavity (arrowheads) (scale bar, 100 μm). (e) A 500 μm *z*‐projection showing CGRP‐labeled nerve axons traversing through the cortical bone (arrow) and following blood vessels (open arrows). Arrowheads indicate examples of nerve endings terminating in the marrow cavity (scale bar, 100 μm). (f) A 250 μm *z*‐projection of a nerve axon entering the marrow cavity from cortical bone (arrow) and terminating in the marrow cavity (arrowheads) (scale bar, 100 μm). (g) Insets show a 3D reconstruction and tracing of a cluster of complex nerve endings indicated in (a) viewed from the front (*XY*‐plane; left) and side (*YZ*‐plane; right). Asterisk indicates the parent axon (scale bars, 100 μm). Dotted lines indicate cortical bone. CB, cortical bone; MC, marrow cavity.

CGRP is a marker of peptidergic sensory neurons. CGRP‐labeled axons entered the marrow cavity via the nutrient foramina at the proximal neck of the femur (Figure 3a,c) or at the distal epiphysis (Figure 3a,b) (Video S3). They ran in parallel down the diaphysis and often followed blood vessels (Figure 3d, open arrows) but did not have the corkscrew morphology observed for some of the nerve fibers labeled with the pan‐neuronal marker, PGP9.5. They typically had numerous *en passant* varicosities along their length (Figure 3d,e) and most often terminated away from blood vessels as a free ending in the marrow cavity or near the endosteum (Figure 3d,e, arrowheads). Some axons followed blood vessels from the marrow cavity and into Volkmann's and Haversian canals in the cortical bone (Figure 3e, arrow), whilst others entered from the cortical bone and terminated in the marrow cavity (Figure 3f). Occasionally, CGRP‐labeled nerve fibers terminated as very complex endings in the lower diaphysis of the marrow cavity (Figure 3g, Video S3). An example filament trace of a CGRP‐labeled complex ending had a total length of ∼7659 μm, with 102 branching points and 65 terminal endings (Figure 3g).

### 3.3 TH‐labeled nerve fibers in the marrow cavity of the femur

**FIGURE 4:**
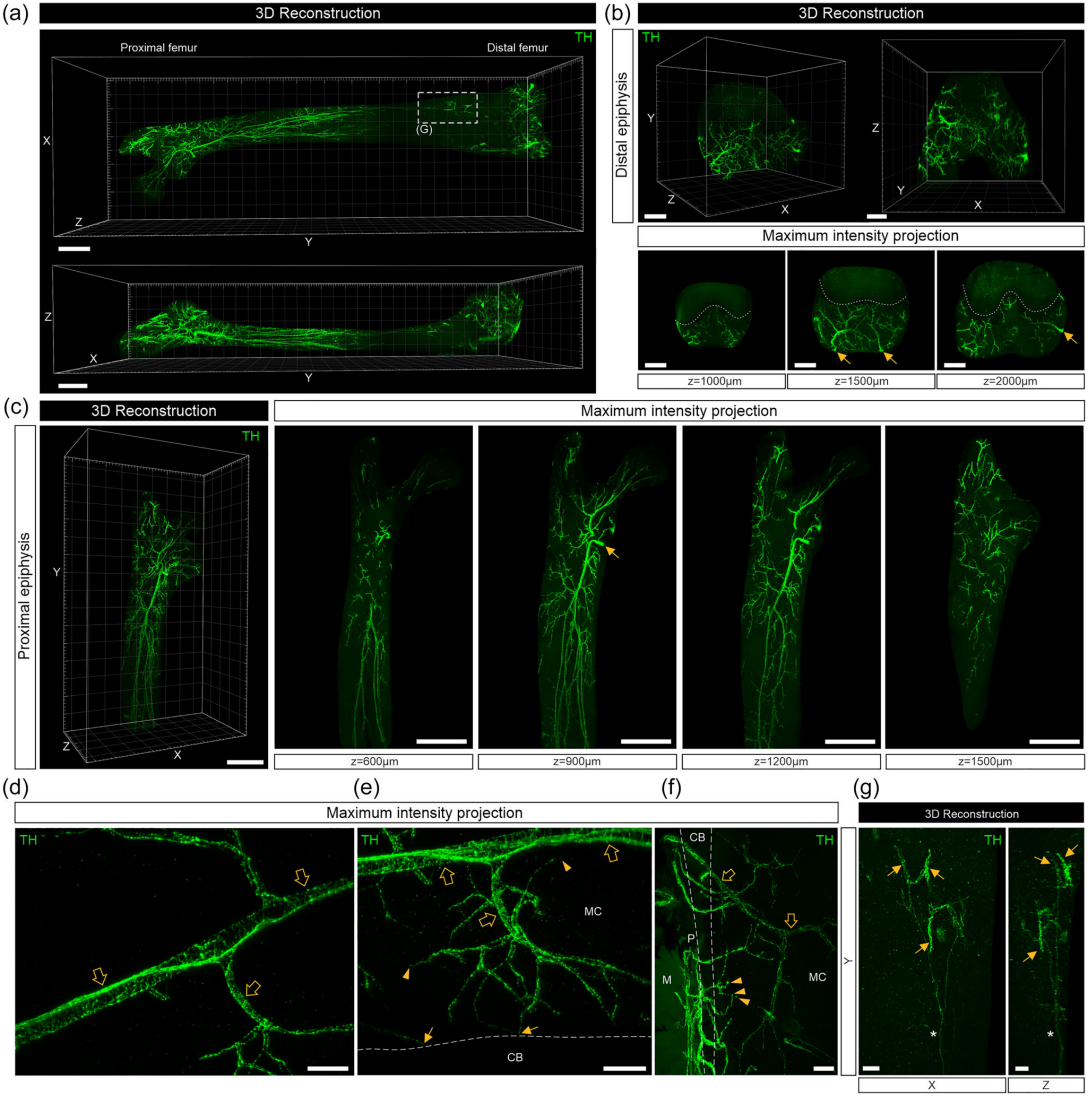
Whole‐tissue immunolabeling and three‐dimensional (3D) fluorescence imaging to visualize tyrosine hydroxylase (TH)‐labeled nerve fibers and their endings in the mouse femur. (a) Mosaic stitched, 3D reconstructed images of the bone marrow after surface rendering (scale bars, 1000 μm). (b, above) 3D reconstructed images of the distal femoral epiphysis. (b, below) Insets show 500 μm maximum intensity *z*‐projections of nerve bundles (arrows) entering the distal epiphysis. Dotted lines indicate the growth plate (scale bars, 500 μm). (c, left) A 3D reconstructed image of the proximal femoral epiphysis and diaphysis. (c, right) Insets show 500 μm *z*‐projections of TH‐labeled nerve bundles (arrow) entering and branching throughout the marrow cavity, mostly clustering around blood vessels (scale bars, 1000 μm). (d) A 200 μm *z*‐projection of TH‐labeled sympathetic nerve fibers imaged at higher power, showing the distinct corkscrew morphology around a blood vessel (open arrows) (scale bar, 100 μm). (e) A 500 μm *z*‐projection of TH‐labeled nerve fibers branching away from blood vessels (open arrows) and terminating at the endosteum near cortical bone (arrows) or in the marrow cavity (arrowheads) (scale bar, 100 μm). (f) A 600 μm *z*‐projection showing TH‐labeled nerve fibers following blood vessels from marrow cavity into cortical bone (open arrows). Some axons entered the marrow cavity from the cortical bone and terminated as free endings in the marrow cavity (arrowheads) (scale bar, 100 μm). (g) Insets show 3D reconstructed images of a single‐TH‐labeled axon indicated in (a) branching and wrapping around multiple blood vessels (arrows) viewed from the front (*XY*‐plane; left) and side (*YZ*‐plane; right). Asterisk indicates the parent axon (scale bar, 200 μm). Dotted lines indicate cortical bone. CB, cortical bone; M, muscle; MC, marrow cavity; P, periosteum.

TH is the rate‐limiting enzyme involved in catecholamine synthesis and a marker for sympathetic noradrenergic nerve fibers. In contrast to the CGRP‐labeled sensory nerve fibers in the femur, TH‐labeling had a distinct corkscrew morphology around blood vessels with relatively few axons branching away to terminate in the marrow cavity or near the endosteum (Figure 4a–f, Video S4). There were many TH‐labeled nerve fibers and endings in the subchondral bone at the distal epiphysis (Figure 4b). TH‐labeled nerve fibers traversed through canals in the cortical bone, following blood vessels from the marrow cavity into cortical bone (Figure 4f, open arrows), or entered and terminated in the marrow cavity from the cortical bone (Figure 4f, arrowheads). On occasion, we observed a very complex branching relationship of TH‐labeled nerve fibers with blood vessels in the lower diaphysis (Figure 4g, Video S4). In these cases, we observed a single axon that branched and wrapped around two or three separate blood vessels in the same area (arrows), with terminal endings closely apposed to the vessels (Figure 4g).

### 3.4 PGP9.5‐immunolabeled nerve fibers in the mandibular tooth pulp and surrounding dental tissue

**FIGURE 5:**
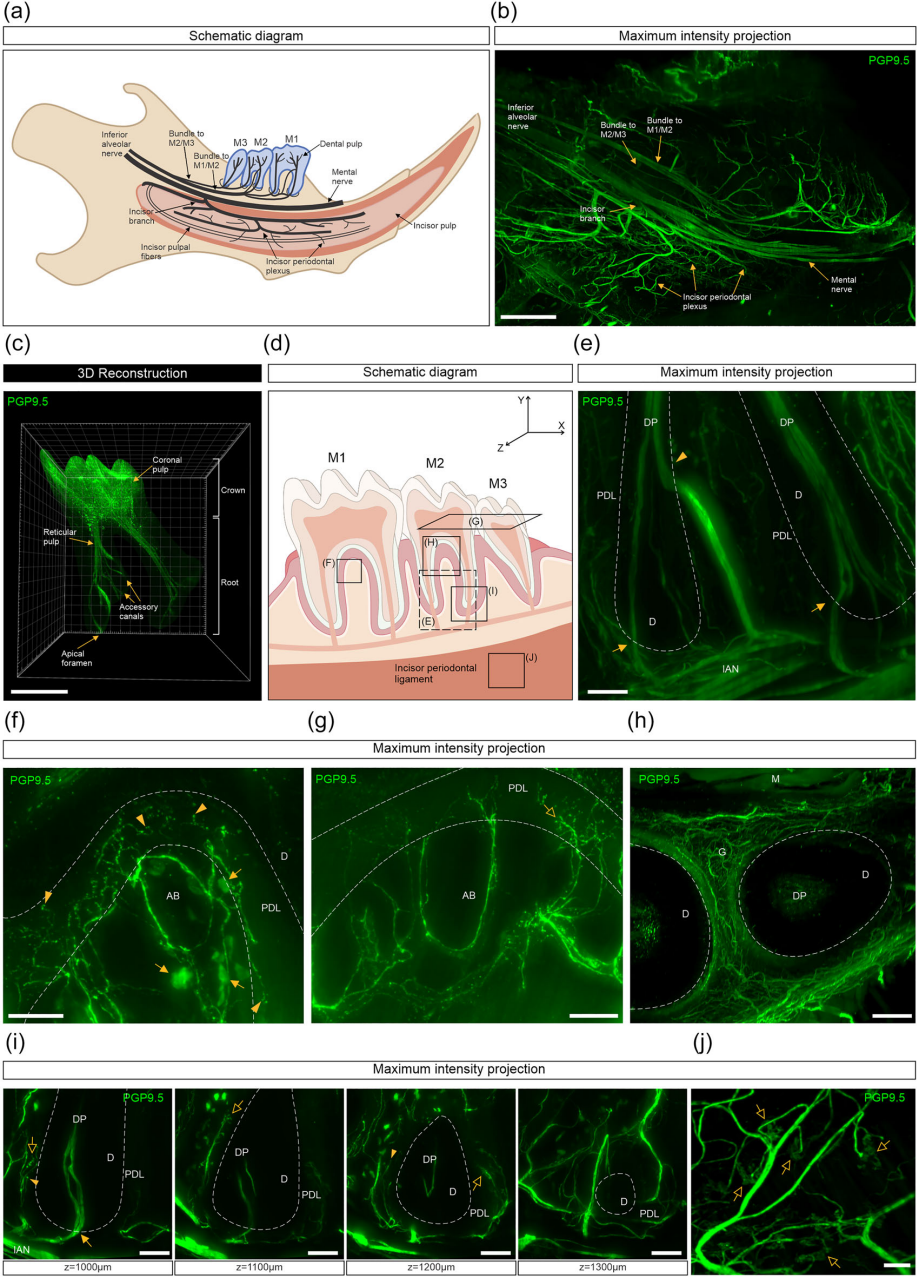
Whole‐tissue immunolabeling and three‐dimensional (3D) fluorescence imaging to visualize protein gene product 9.5 (PGP9.5)‐labeled nerve fibers and their endings in the mouse mandible and surrounding tissues. (a) A simplified schematic representation of the neuronal distribution in the mouse mandible. (b) An 800 μm maximum intensity *z*‐projection showing the distribution of inferior alveolar nerve branches through the mandible (scale bar, 500 μm). (c) A 3D‐reconstructed image of the dental pulp after surface rendering (scale bar, 500 μm). (d) Schematic representation indicating the location from which e‐j were sampled and viewed. (e) A 400 μm *z*‐projection showing a bundle of nerve fibers that entered the molar through the apical foramen (arrows) and an accessory canal (arrowhead) (scale bar, 100 μm). (f) A 150 μm *z*‐projection showing nerve fibers running through the mandibular alveolar bone, including the marrow, and nerve terminal endings in the periodontal ligament (arrowheads). Arrows indicate PGP9.5‐labeled cells in the alveolar bone (scale bar, 100 μm). (g) An 80 μm *z*‐projection showing nerve fibers running through the mandibular alveolar bone, including the marrow, and complex Ruffini‐like endings in the periodontal ligament (open arrow) (scale bar, 100 μm). (h) A 100 μm *z*‐projection showing innervation of the gingiva around the tooth (scale bar, 200 μm). (i) 100 μm *z*‐projections showing a PGP9.5‐labeled nerve bundle entering the apical foramen (arrow) or branching and terminating around the periodontal ligament in a “basket”‐like manner around the molar root. The periodontal nerve fibers terminated as free endings (arrowheads) or Ruffini‐like endings (open arrows) (scale bars, 100 μm). (j) A 100 μm *z*‐projection showing complex Ruffini‐like endings (open arrows) in the incisor periodontal ligament (scale bar, 500 μm). AB, alveolar bone; D, dentin; DP, dental pulp; G, gingiva; IAN, inferior alveolar nerve; M, muscle; PDL, periodontal ligament.

The mouse mandible consists of three molars and one continuously growing incisor in each half of the jaw (Figure 5a). The PGP9.5‐labeled nerve fibers that innervate the molars emerge as bundles from the inferior alveolar nerve, running in parallel and splitting into smaller branches as they approach the molars and their surrounding tissue (Figure 5a,b, Video S5). Each of these small bundles gives off branches that entered the dental pulp through the apical foramen of the molar roots (Figure 5c–e,i). These nerve fibers projected through the dental root to the coronal pulp where they split and branched extensively to form a dense plexus (Figure 5c, Video S5). We noted another bundle of nerve fibers entering the first (M1) and second (M2) molars, higher up the dental root through an accessory canal, and into the main radicular pulp (Figure 5e, arrowhead) or even directly into the coronal pulp. Some small branches gave off axons that terminated as free, but not complex, endings in the mandibular alveolar bone, including in marrow, around the roots of the molars (Figure 5f,g). Other branches entered the periodontal ligament (Figure 5f,g) or gingiva (Figure 5h) where they terminated as either free fiber endings, or more complex Ruffini‐like endings (Figure 5f,g,i). The branches that innervated the periodontal ligament formed “basket”‐like structures around the root of the molars (Figure 5i). Some of these endings had varicosities along their length and some did not. The inferior alveolar nerve continued as the mental nerve, and the incisor nerve sent branches to the incisor periodontal plexus and pulp (Figure 5a,b). Ruffini‐like endings were also observed in the periodontal ligaments around the incisor (Figure 5j). On occasions, we noted that the PGP9.5 antibody labeled cell‐like structures in the alveolar bone or periodontal ligament around the molar root (Figure 5f, arrows).

### 3.5 CGRP‐immunolabeled nerve fibers in the mandibular tooth pulp and surrounding dental tissue

**FIGURE 6:**
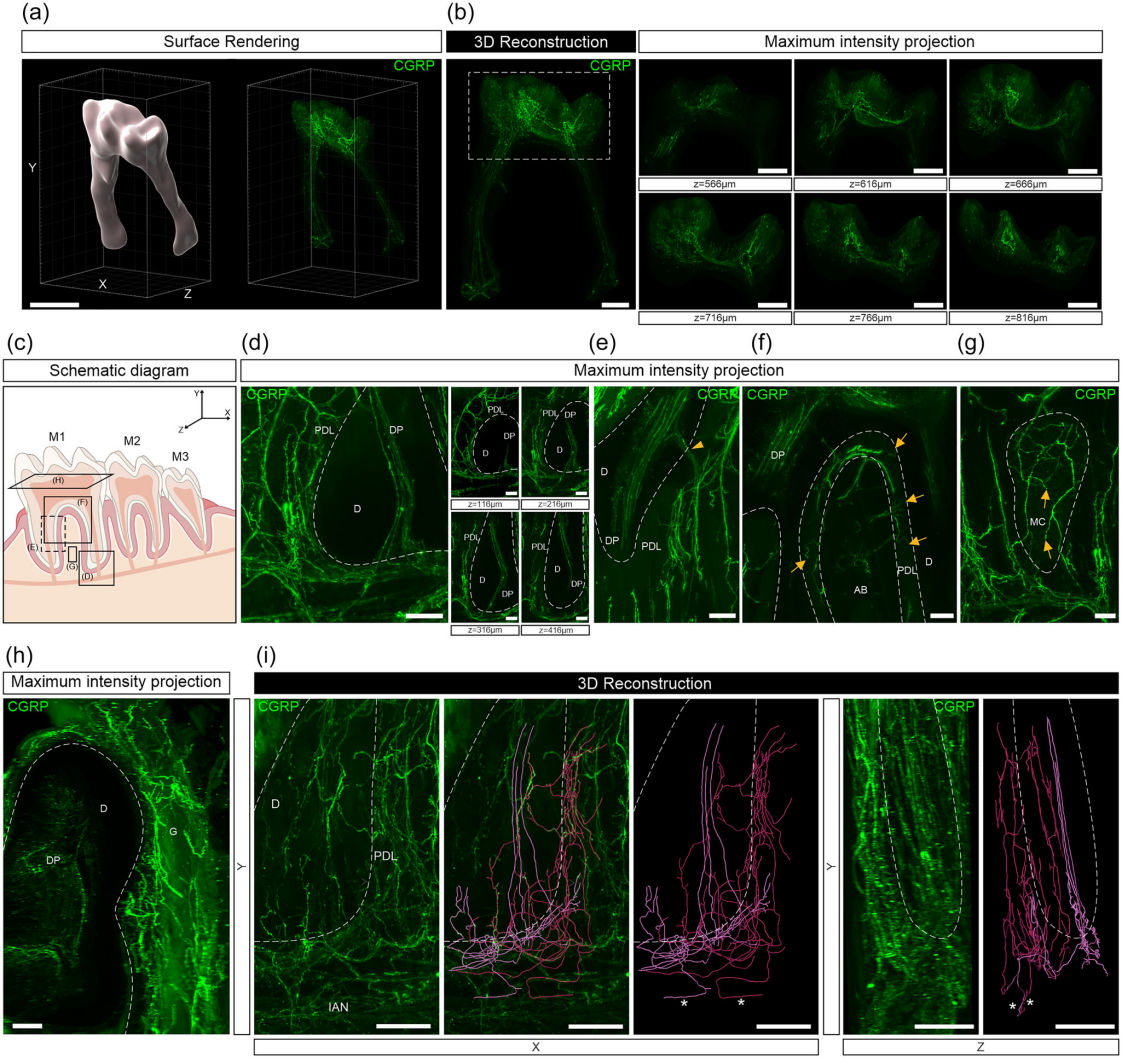
Whole‐tissue immunolabeling and three‐dimensional (3D) fluorescence imaging to visualize calcitonin gene–related peptide (CGRP)‐labeled nerve fibers and their endings in the mouse mandible and surrounding tissues. (a) 3D reconstructed images of the dental pulp after surface rendering (scale bar, 500 μm). (b, left) A 3D‐reconstructed image of the dental pulp. (b, right) Insets show 50 μm maximum intensity *z*‐projections of nerve fibers through the coronal pulp indicated in the dotted square (scale bar, 200 μm). (c) Schematic representation indicating the location from which (d–h) were sampled and viewed. (d, left) A 400 μm *z*‐projection of the molar root showing sensory nerve axons entering the apical foramen, or branching to the surrounding tissue where they form “baskets” around the molar root. (d, right) Insets show 200 μm *z*‐projections through the molar root (scale bars, 100 μm). (e) A 300 μm *z*‐projection showing another branch of nerve axons entering the first molar higher in the root through an accessory canal (arrowhead) (scale bar, 100 μm). (f) A 100 μm *z*‐projection showing terminal endings (arrows) in the periodontal ligament around the molar root (scale bar, 100 μm). (g) A 200 μm *z*‐projection showing terminal endings in the mandibular alveolar bone, including the marrow (scale bar, 50 μm). (h) A 200 μm *z*‐projection showing sensory innervation of the gingiva around the tooth (scale bar, 100 μm). (i) 3D reconstruction and tracing of two separate axons with multiple branching and terminal endings viewed from the front (*XY*‐plane; left) and side (*YZ*‐plane; right). Asterisk indicates a single axon (scale bar, 100 μm). AB, alveolar bone; D, dentin; DP, dental pulp; G, gingiva; MC, marrow cavity; PDL, periodontal ligament.

There was extensive innervation by CGRP‐labeled peptidergic sensory neurons in the dental pulp and surrounding tissues (Figure 6a,b, Video S6). The CGRP‐labeled axons that entered the dental pulp did so through both the apical foramen (Figure 6d) and the accessory canal (Figure 6e) and provided a very dense innervation throughout the entire pulp (Figure 6b). CGRP‐labeled axons also branched extensively into the periodontal ligament (Figure 6f), mandibular alveolar bone, including marrow (Figure 6g), and/or gingiva (Figure 6h), where they mostly terminated as free nerve endings. In many cases, we were able to resolve the nerve terminal structure of individual CGRP‐labeled axons in the periodontal ligaments and found they terminated as free endings that contributed to the “baskets” around each molar (Figure 6i, Video S7). CGRP‐labeled axons did not appear to have the same complexity as some of the PGP9.5‐labeled endings and did not contribute to Ruffini‐like endings.

### 3.6 TH‐immunolabeled nerve fibers in the mandibular tooth pulp and surrounding dental tissue

**FIGURE 7:**
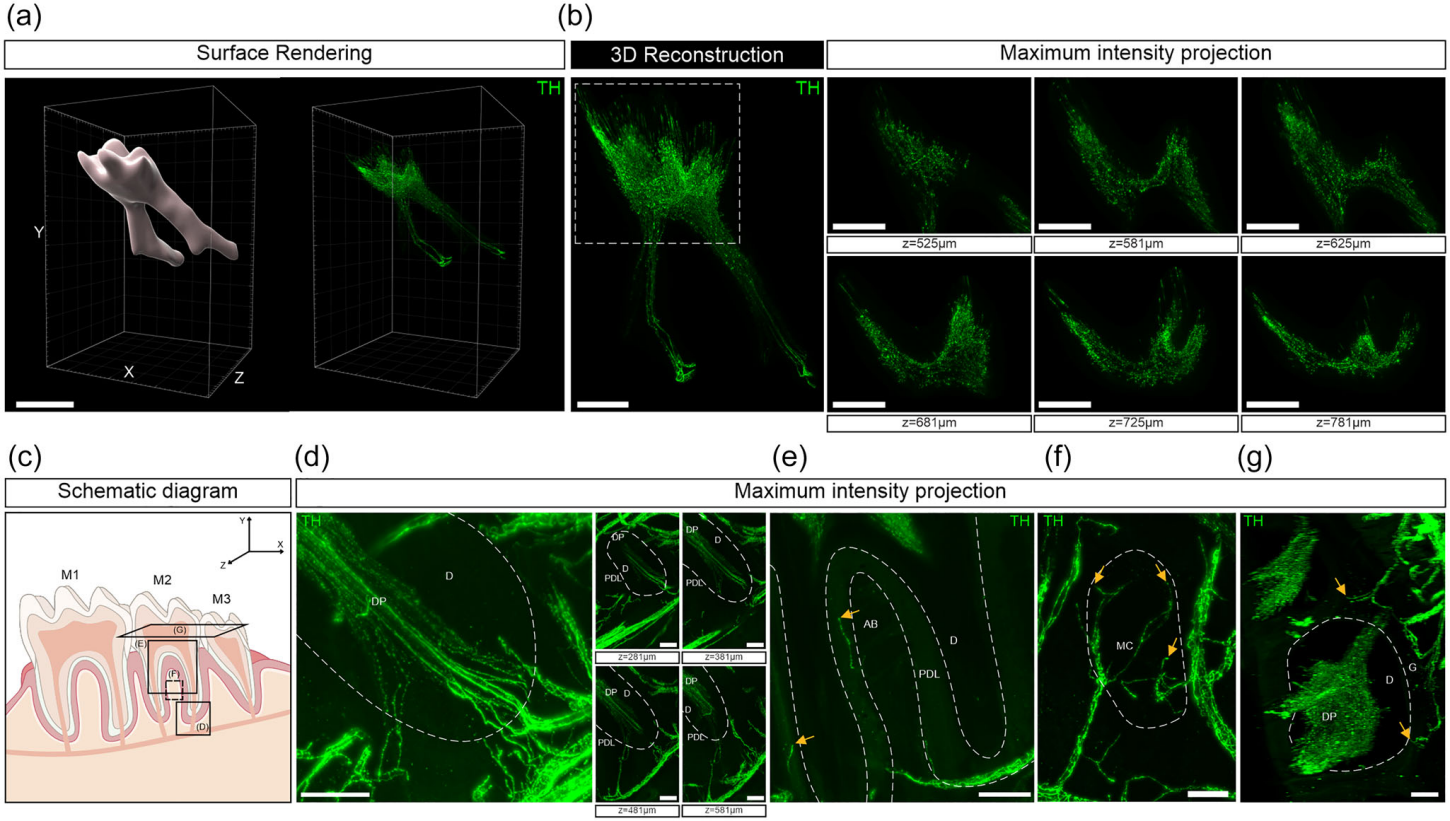
Whole‐tissue immunolabeling and 3D fluorescence imaging to visualize tyrosine hydroxylase (TH)‐labeled nerve fibers and their endings in the mouse mandible and surrounding tissue. (a) 3D reconstructed images of the dental pulp after surface rendering (scale bar, 500 μm). (b, left) A 3D‐reconstructed image of the dental pulp. (b, right) Insets show 20 μm maximum intensity *z*‐projections of nerve fibers through the coronal pulp indicated in the dotted square (scale bar, 200 μm). (c) Schematic representation indicating the location from which (d–g) were sampled and viewed. (d) A 600 μm *z*‐projection of the molar root showing TH‐labeled nerve axons entering through apical foramen and into the dental pulp. (d, right) Insets show 300 μm *z*‐projections through the molar root (scale bar, 100 μm). (e) A 100 μm *z*‐projection showing few terminal endings (arrows) in the periodontal ligament around the molar root (scale bar, 100 μm). (f) A 300 μm *z*‐projection showing terminal endings in the marrow of mandibular alveolar bone (scale bar, 100 μm). (g) A 400 μm *z*‐projection showing sympathetic innervation of the gingiva around the tooth. Arrows indicate nerve terminal endings (scale bar, 100 μm). AB, alveolar bone; D, dentin; DP, dental pulp; G, gingiva; MC, marrow cavity; PDL, periodontal ligament.

The dental pulp was also heavily innervated by fine TH‐labeled nerve fibers and endings (Figure 7a,b, Video S8). The TH‐labeled axons that entered the dental pulp did so through both the apical foramen and the accessory canal (Figure 7d), although the number of axons that we observed entering the accessory canal were much fewer than those labeled with antibodies directed against CGRP (Figure 7 is an example). In some cases, there were no TH‐labeled nerve fibers in the nerve that entered the accessory canal. In the surrounding tissue, TH‐labeled nerve fibers were mostly perivascular, spiraling around vessels in a manner similar to what was observed in the femur (Figure 7d,f, Video S8). The incisor periodontal plexus, molar periodontal ligaments (Figure 7e), mandibular alveolar bone, including marrow (Figure 7f), and gingiva (Figure 7g) were sparsely innervated by TH‐labeled nerve fibers. Only few of the TH‐labeled axons in these tissues terminated as free endings away from blood vessels (Figure 7e–g), and they did not contribute to the “basket”‐like structures around the roots of the molars (Figure 7d).

## 4 DISCUSSION

In this study, we have modified the iDISCO/iDISCO+ tissue clearing protocol to label peripheral nerve fibers, and their terminal endings, in intact whole bone and dental tissues in adult mice. Nerve fibers that innervate long bone and dental tissue have multiple branches and terminal endings and often spread over long distances, making them difficult to follow in traditional histological sections. The protocol outlined here allows for the 3D visualization of nerves in hard tissues and is easily applicable to studies of murine long bone and dental tissues.

### 4.1 Advances in tissue clearing and imaging of hard tissue

Recent advances in optical clearing techniques (e.g., CLARITY, DISCO series, CUBIC, and PEGASOS) and light sheet fluorescence microscopy have enabled the 3D imaging of cellular structures in intact tissues and organs (Jensen & Berg, 2017; Molbay et al., 2021; Susaki et al., 2015). Whilst 3D imaging of both human and mouse long bones (Greenbaum et al., 2017; Gruneboom et al., 2019; Jing et al., 2018; Lorenz et al., 2021; Utagawa et al., 2023; Wang et al., 2019) and teeth (Franca et al., 2019; Hong et al., 2019) has recently been achieved, most studies have relied on transgenic fluorescence or in vivo antibody delivery to detect vasculature, neuronal, and cellular structures. An excellent recent example of this is the work of Utagawa et al. (2023), who developed a novel approach that they referred to as Osteo‐DISCO, to preserve endogenous fluorescence and enable 3D reconstruction of nerve bundles through a number of different long bones and the jaw. However, the authors of this study reported nerve bundles in *Sox10‐Venus* transgenic reporter mice, which label mature and immature oligodendrocytes and Schwann cells, not neurons, so the labeling they observed is not neuronal (Utagawa et al., 2023). In addition, Schwann cells are mostly located around axons in nerve bundles and do not extend to and encase the most distal parts of nerve terminal endings, so the authors did not report the fine detail of their structure, or how individual axons interact with their target at their endings. Whilst they attempted to address this by immunolabeling sensory and sympathetic nerve fibers, and their endings, with antibodies directed against CGRP and TH, respectively, this was done only on histological sections (Utagawa et al., 2023). Another study using the tissue clearing method BoneClear showed robust immunolabeling of PGP9.5 and TH in the mouse femur and hind paw but did not describe the distribution of labeled nerves through the different parts of long bones or in the jaw (Cao & Yang, 2020; Wang et al., 2019). There is a clear need for further development of optical clearing procedures for intact bone and dental tissues that are compatible with whole mount immunolabeling.

Here, we report a modified iDISCO/iDISCO+ clearing protocol for the 3D imaging of nerve endings immunolabeled with sensory and sympathetic neuronal markers that innervated different compartments of the mouse femur and mandible. It provides unprecedented insight into the neuroanatomy and distribution of these neurons that was not possible in the past.

### 4.2 Innervation of the mouse femur

In this study, we report high‐quality immunolabeling of the axons and nerve terminal endings of sensory and sympathetic neurons that innervate the mouse femur. Importantly, we were able to follow both sensory and sympathetic axons, and their nerve terminal endings, all the way through the femur, highlight how extensive their distribution is, and show regional differences in innervation patterns for different parts of the bone.

Nerve bundles containing both sensory and sympathetic axons entered the marrow cavity of the femur via nutrient foramina located at the proximal metaphysis and the distal epiphysis of the femur. The nerves that entered the marrow cavity at the proximal end of the femur branched extensively into smaller bundles that ran in parallel and terminated over long distances, some extending all the way down to the distal metaphysis. They often followed blood vessels through the marrow cavity. These findings for the distribution of nerve bundles in the mouse femur are consistent with previous studies, which have used *Sox10‐Venus* reporter mice to identify the nerves by labeling the supporting cells around axons within nerve bundles (Utagawa et al., 2023). Because we were able to label axons distal to the point at which they lost their supporting Schwann cells, we further add significant detail relating to the distribution of nerve terminal endings within the bone. For example, CGRP‐labeled nerve fibers and endings often followed blood vessels but did not have the corkscrew morphology observed for some of the PGP9.5‐labeled fibers and often terminated away from blood vessels as free endings in the marrow cavity or near the endosteum. In contrast, TH‐labeled nerve fibers and endings typically spiraled around blood vessels and less frequently terminated as free endings away from blood vessels. We were also able to clearly demonstrate numerous *en passant*–type varicosities along the length of nerve axons and follow them all the way to their endings where they interact with their target tissue. Whilst we do not have the resolution to identify contacts with target cells if they do indeed exist, we were able to show differential distribution patterns and morphology for sensory and sympathetic neurons in different parts of the bone. Mapping the distribution of these endings in different bony compartments may be important in further elucidating their roles during homeostasis, and in injury or disease.

Our finding of clusters of very complex endings associated with the termination of a single axon in the mid‐to‐lower diaphysis is highly novel. These types of endings are not reported in bony tissue as images taken from single histological sections can often miss these complex structures. CGRP‐labeled complex endings were highly branched structures at the end of a long axon and were consistently located in the mid‐to‐lower third of the femur. Whilst it is difficult to ascribe a function to these complex CGRP‐labeled sensory endings in bone, similar clusters of short nerve terminal branches have been described at the receptive sites of vagal and dorsal root ganglion mechanoreceptors in the intestine (Lynn et al., 2005, 2003; Zagorodnyuk & Brookes, 2000), lung (Mazzone & Undem, 2016; Su et al., 2022; Wang et al., 2017), bladder (Zagorodnyuk et al., 2009), and muscle (Jankowski et al., 2013). Whether these complex endings are mechanoreceptive and can contribute to mechanical load sensing requires further attention. We also observed TH‐labeled complex endings that often ended by branching into two or three distinct endings that wrapped around several small vessels in close apposition to each other, perhaps suggesting a highly localized regulation of the vasculature by sympathetic neurons in the bone marrow. Hematopoietic stem cell (HSC) niches may also be relevant to consider here, as they are highly localized and there is emerging evidence for a role for the sympathetic nervous system in HSC trafficking and quiescence (Golan et al., 2018; Katayama et al., 2006; Maryanovich et al., 2018; Mendez‐Ferrer et al., 2008; Yamazaki et al., 2011).

Our finding that axons that run down through the diaphysis of the femur gave branches that projected laterally, toward the endosteum and terminating near cortical bone, has a number of significant implications. We speculate that they may be in contact with osteoprogenitor cells and/or specialized bone cells in the endosteum and could therefore be involved in regulating bone homeostasis. Indeed, several studies have suggested that bone marrow stromal cells (the primary source of osteoprogenitor cells), and osteoblasts, express CGRP receptors in vitro (Wang et al., 2010), and that CGRP can promote osteoblast differentiation, proliferation, and mineralization (Bjurholm et al., 1992; Jia et al., 2019; Li et al., 2021; Wang et al., 2010). Other studies have shown that there are TH‐labeled fibers found in the vicinity of osteoblasts and that these cells, and osteocytes, express β_2_‐adrenergic receptors (Elefteriou et al., 2005; Imai & Matsusue, 2002; Takeda et al., 2002; Zhu et al., 2018). Animal studies have also demonstrated clear evidence for roles for these neurons in regulating bone metabolism (Ballica et al., 1999; Ding et al., 2010; Jia et al., 2019; Offley et al., 2005; Schinke et al., 2004; Takeda et al., 2002).

Our results also support previous studies that have reported a decreased innervation density for both CGRP‐ and TH‐labeled axons and nerve terminal endings lower in the femur (Castaneda‐Corral et al., 2011; Chartier et al., 2018; Fujita et al., 2022; Mach et al., 2002). In healthy adult bone, areas of high osteogenic capacity often display the highest density of sensory and sympathetic fibers (Bjurholm, Kreicbergs, Brodin, et al., 1988; Mach et al., 2002; Sisask et al., 2013; Steverink et al., 2021). The proximal part of the femur is often subjected to significant mechanical loads and compression (Kersh et al., 2018; Lotz et al., 1995) and has high metabolic activity and bone turnover (Bagi et al., 1996, 1997), which may explain the higher density of nerve fibers observed in this area.

### 4.3 Innervation of the mouse mandible and dental tissues

In this study, we report high‐quality immunolabeling of the axons and nerve terminal endings of sensory and sympathetic neurons in the mouse mandible. We were able to follow both sensory and sympathetic axons, and their nerve terminal endings, from the inferior alveolar nerve and into the dental pulp of the molar teeth and surrounding tissues. We were able to highlight how extensive their distribution is and show regional differences in the innervation patterns for different neuronal subpopulations.

There have been numerous studies that have documented in detail the innervation of the tooth pulp and dentin (Aker, 1987; Byers, 1985; Byers et al., 2003; Corpron & Avery, 1973; Fearnhead, 1957, 1961; Hildebrand et al., 1995; Johansson et al., 1992; Kato et al., 1990; Luthman et al., 1992; Naftel et al., 1999; Sato et al., 1988). Our findings of a dense sensory and sympathetic innervation of the tooth pulp are consistent with numerous other studies that have reported CGRP‐labeled peptidergic sensory axons and TH‐labeled sympathetic axons in dental tissue (Heyeraas et al., 1993; Moe et al., 2008; Rodd & Boissonade, 2002; Uddman et al., 1986). Many of these previous studies have found that sensory and sympathetic nerve endings terminate near fibroblasts, odontoblasts/osteoclasts, and endothelial cells, that each express receptors for neuropeptides to regulate bone metabolism and blood flow (Bongenhielm et al., 1995; Calland et al., 1997; Elefteriou et al., 2005; Ibuki et al., 1996; Shimeno et al., 2008; Zhang & Fukuyama, 1999). Peptidergic sensory neurons also have well‐established roles in generating and maintaining pain associated with injury and pathology. Sensory neurons in the dental pulp are predominately small‐diameter thinly myelinated (Aδ‐fiber) or peptidergic unmyelinated (C‐fiber) nociceptors, and stimulation of the exposed pulp and dentine, even to weak mechanical stimuli like air‐puffs and hydrostatic pressure, primarily evokes pain (Byers & Narhi, 1999; Cadden et al., 1983; Charoenlarp et al., 2007; Fried et al., 1988, 2011; Narhi et al., 1994, 1982; Vongsavan & Matthews, 2007).

Interestingly, whilst we have noted that CGRP‐ and TH‐labeled axons mostly enter the dental pulp through the apical foramen of the molar root, we also reported an additional and consistent entry point for these axons into the radicular pulp via an accessory canal higher up the root. Early studies have identified these canals in human teeth (Ahmed et al., 2018; De Deus, 1975; Green, 1955; Hildebrand et al., 1995; Ringelstein & Seow, 1989; Vertucci, 1984), and although they have been extensively studied in the clinic, little is known about how they transmit nerves to and from the pulp. One study mentioned that in human premolars, the accessory canal to the coronal pulp contained small‐to‐large nerve bundles and fibers (Franca et al., 2019). Here we show a thick PGP9.5‐labeled nerve bundle that enters an accessory canal in all samples of mouse molars. It contains numerous CGRP‐labeled sensory axons and few or no TH‐labeled fibers. With whole mandible imaging, we can confirm that this is a branch of the inferior alveolar nerve that also supplies the apical foramen and comes up through the alveolar bone and periodontal ligament before entering the dental pulp. Sensory and sympathetic fibers first enter the dental pulp at different stages of tooth development, suggesting that combinations of locally expressed signaling molecules strictly control the pattern and timing of penetration of sensory and sympathetic fibers into teeth (Kettunen et al., 2005; Luukko et al., 1996; Moe et al., 2008). This may help explain the differential distribution of CGRP‐ and TH‐labeled fibers through the apical and accessory canals into the dental pulp.

We also observed a differential distribution of CGRP‐ and TH‐labeled fibers in the periodontal tissue that surrounds the molars. CGRP‐labeled fibers appeared to densely innervate the periodontal ligament, mandibular alveolar bone, including marrow, and gingiva. In line with previous studies, some CGRP‐labeled fibers appeared to have no obvious relation to blood vessels and terminated as free fiber endings in the surrounding periodontal tissue (Byers, 1985; Heyeraas et al., 1993; Luthman et al., 1992; Uddman et al., 1984; Wakisaka et al., 1987). CGRP released from peptidergic C‐fibers in response to mechanical stress is immediate, increases the sensitivity of nociceptors by triggering the release of inflammatory mediators, and may lead to root resorption if the stress is sustained (Caviedes‐Bucheli et al., 2022, 2008; Vandevska‐Radunovic, 1999). In the present study, we also found that the CGRP‐labeled free endings that innervated the periodontal ligament were organized in a “basket”‐like manner around the roots of the molar. Often, a few branches of the inferior alveolar nerve contribute to the baskets around each root, with the terminal field of each branch overlapping significantly with the terminal field of other branches. Whilst this suggests some redundancy in function, it might also provide opportunity for the sprouting of neighboring branches into the periodontal ligaments after injury and may be relevant in the context of changes that occur after dental manipulation (e.g., tooth extraction). Indeed, there is some evidence that tooth movement increases the density of CGRP‐labeled fibers, suggesting an involvement for peptidergic sensory neurons in early stages of periodontal remodeling, and later in regenerative processes (Norevall et al., 1995; Saito et al., 1991; Vandevska‐Radunovic & Murison, 2010; Vandevska‐Radunovic et al., 1997). In contrast, TH‐labeled fibers were mostly perivascular and have a clear role in vasoregulation (Heyeraas et al., 1993; Luthman et al., 1992; Uddman et al., 1986, 1984). In the present study, there was only sparse innervation of the periodontal ligament, mandibular alveolar bone, including marrow, and gingiva by TH‐labeled nerve endings, and they did not contribute in a significant way to the “baskets” we observed in the periodontal tissue. It will be interesting to use this approach to visualize periodontal “baskets” in 3D to explore if and how they change in response to disease, experimental dental manipulation, or injury to the dental innervation itself.

### 4.4 Optimization of the protocol and recommendations for implementation

The modified iDISCO/iDISCO+ protocol reported here produces macroscopically transparent mouse bone and dental tissues and enables 3D visualization of peripheral nerve terminal endings that innervate bone and dental tissues. Here we present a discussion of some of the important steps required for optimization and implementation of the protocol reported in this study.

In preliminary experiments, we evaluated the effect of different decalcification and immunolabeling protocols on the quality of immunolabeling and tissue integrity (Table 2). We found that perfusion fixation and decalcification with Morse's solution at room temperature, for at least 30 h, provided strong immunosignals and optimal deep‐tissue imaging while preserving tissue integrity (Table 2).

**TABLE 2:** The effect of different decalcification methods on staining and structural integrity of bone and dental tissue.

Decalcifying solutions	Processing time	Processing temperature	Bone		Tooth	
			IF	SI	IF	SI
10% EDTA	7 days^a^	4°C	P	P	P	G
5% formic acid	24 h	4°C	–	–	F	E
10% formic acid	24 h	4°C	P	E	F	E
10% formic acid	24 h	RT	P	E	F	E
Morse's solution (22.5% formic acid, 10% tri‐sodium citrate)	24 h	4°C	F	E	G	E
Morse's solution (22.5% formic acid, 10% tri‐sodium citrate)	30–40 h	RT	E	E	E	E
Morse's solution (45% formic acid, 10% tri‐sodium citrate)	24 h	4°C	F	P	–	–

Abbreviations: E, excellent; EDTA, ethylenediaminetetraacetic acid; F, fair; G, good; IF, immunofluorescence; P, poor; RT, room temperature; SI, structural integrity.

^a^Solution changed every second day.

Our protocol is reliant on the identification of antibodies that are compatible with the methanol pre‐treatment and can penetrate deep into bone. We recommend different antibodies are first tested on decalcified tissue sections and incubating the sections with methanol before immunostaining. This will provide an important positive control for the use of the antibody in large‐volume samples. It is also important to trial different concentrations for a given antibody, and different blocking, antibody incubation and/or decalcification times.

We often observed autofluorescence of mineralized tissue, even after decalcification, which obscured imaging of fine nerve terminals deeper in the sample. This is particularly evident for the CGRP and PGP9.5 antibodies used. Following light sheet microscopy, images were post‐processed using Imaris to render a 3D surface at the interface between soft bone marrow or dental pulp tissue, and mineralized tissue, allowing us to digitally remove/mask the dense bone that produced tissue autofluorescence and provide an unobstructed view of the nerve terminal endings deep within hard tissue (Figure 1, Videos S1 and S5). In addition, we found tissue autofluorescence could be further minimized by ensuring the animal is completely cleared of blood during perfusion, reducing post‐fixation time, increasing blocking times, and using fluorophores with longer emission wavelength.

The quality of imaging has a significant impact on the outcome. In our preliminary experiments, we often experienced light streaks through the sample, images that were under or oversaturated, background that was too high, and/or blurry images. To overcome these issues, we made sure that muscles/surrounding tissues were removed as much as possible during processing, that decalcification was complete, and that there were no bubbles or lint inside or around the tissue sample during acquisition. We also modified the acquisition parameters of the microscope, including the laser intensity, light sheet numerical aperture, thickness of the light sheet, and exposure time to obtain optimal images. Blurred images can often be caused by poor perfusion or insufficient delipidation, so it might also be important to increase DCM incubation time and ensure samples have sunk in DCM.

## 5 FUTURE DIRECTIONS

In this study, we have described a modified iDISCO/iDISCO+ protocol that can be used for the 3D visualization of nerves in hard tissues and is easily applied to studies of murine long bone and dental tissue. Our investigations, however, were confined to the labeling of axons and nerve terminal endings of a few specific neuronal subpopulations in the mandible and dental tissues, and the long bones of the lower limb. It will be interesting in the future to explore the distribution of other important subpopulations of neurons in these, and other hard tissues throughout the body. For example, sensory neurons in the periodontal tissue mediate both painful sensations and touch and pressure in the periodontal space. In the present study, we have clearly identified free nerve endings of peptidergic sensory neurons that are likely to be involved in painful sensation, but it will be important in the future to also better define the molecular phenotype and structure of the more complex Ruffini endings that are responsible for innocuous mechanosensitivity in periodontal tissues (Byers, 1985; Kannari, 1990; Kannari et al., 1991; Trulsson, 2006; Trulsson et al., 1992). In addition, cholinergic innervation has been reported in the periosteum (Anderson et al., 2006; Asmus et al., 2001), bone marrow (Fielding et al., 2022; Gadomski et al., 2022), and subchondral bone (Courties et al., 2020). It would be interesting to further identify how these cholinergic nerves are distributed, and how closely they associate with the cells that they might interact with, throughout the different tissue compartments in bone. These could easily be facilitated with further modifications to the protocol that combine the use of double‐label immunohistochemistry and/or murine genetic reporter lines. We have also demonstrated that the quality of the labeling that can be achieved with this approach enables filament tracing of axons and nerve terminals through hard tissues, an approach that will be important to use in future studies exploring how the innervation of hard tissues changes in models of disease or pathology.

## CONFLICT OF INTEREST STATEMENT

The authors declare no conflicts of interest.

## Supporting information


**Video S1** Whole‐tissue imaging and 3D segmentation of the marrow cavity of a mouse femur immunolabeled with an anti‐PGP9.5 antibody. This movie corresponds to Figure 2B–D and shows neuronal distribution through the marrow cavity.

**Video S2** Whole‐tissue imaging of a mouse mandible immunolabeled with an anti‐PGP9.5 antibody and filament traced with Imaris. This 3D movie corresponds to Figure 2G and shows a cluster of complex nerve endings.

**Video S3** Whole‐tissue imaging and 3D segmentation of the marrow cavity of a mouse femur immunolabeled with an anti‐CGRP antibody. This movie corresponds to Figure 3A–C and shows the distribution of sensory neurons through the marrow cavity. A cluster of complex nerve endings was also observed and traced on Imaris, corresponding to Figure 3G.

**Video S4** Whole‐tissue imaging and 3D segmentation of the marrow cavity of a mouse femur immunolabeled with an anti‐TH antibody. This movie corresponds to Figure 4A–C and shows the distribution of sympathetic neurons through the marrow cavity. TH‐labeled axons are often wrapped around blood vessels in a unique corkscrew morphology.

**Video S5** Whole‐tissue imaging of a mouse mandible immunolabeled with an anti‐PGP9.5 antibody and 3D segmentation of dental pulp in the molar. This 3D movie corresponds to Figure 5B and shows the distribution of the inferior alveolar nerve branches through the mandible.

**Video S6** Whole‐tissue imaging of a mouse mandible immunolabeled with an anti‐CGRP antibody and 3D segmentation of dental pulp in the molar. This 3D movie corresponds to Figure 6 and shows dense innervation of sensory neurons in and around the tooth.

**Video S7** Whole‐tissue imaging of a mouse mandible immunolabeled with an anti‐CGRP antibody and filament traced with Imaris. This 3D movie corresponds to Figure 6h and shows single axons with multiple branching and terminal endings around the molar root.

**Video S8** Whole‐tissue imaging of a mouse mandible immunolabeled with an anti‐TH antibody and 3D segmentation of dental pulp in the molar. This 3D movie corresponds to Figure 7 and shows dense innervation of sympathetic neurons in the dental pulp, but less so in the periodontal ligament and gingiva.

## Data Availability

The data that support the findings of this study are available from the corresponding author upon reasonable request.
